# Bis(2,6-dimethyl­pyridinium) hexa­chlorido­platinate(IV)

**DOI:** 10.1107/S1600536808025257

**Published:** 2008-08-09

**Authors:** Vahid Amani, Rahmatollah Rahimi, Hamid Reza Khavasi

**Affiliations:** a Department of Chemistry, Shahid Beheshti University, Tehran 1983963113, Iran; b College of Chemistry, Iran University of Science and Technology, Tehran 16846-13114, Iran

## Abstract

The asymmetric unit of the title compound, (C_7_H_10_N)_2_[PtCl_6_], contains one independent protonated 2,6-dimethyl­pyridinium cation and half of a centrosymmetric [PtCl_6_]^2−^ anion. The Pt atom has an octa­hedral coordination. In the crystal structure, inter­molecular N—H⋯Cl and C—H⋯Cl hydrogen bonds result in the formation of a supra­molecular structure. There is a π–π contact between the pyridine rings [centroid–centroid distance = 4.235 (1) Å].

## Related literature

For related literature, see: Abedi *et al.* (2008 ▶); Bencini *et al.* (1992 ▶); Bokach *et al.* (2003 ▶); Bowmaker *et al.* (1998 ▶); Ciccarese *et al.* (1998 ▶); Delafontaine *et al.* (1987 ▶); Effendy *et al.* (2006 ▶); Hasan *et al.* (2001 ▶); Hojjat Kashani *et al.* (2008 ▶); Hu *et al.* (2003 ▶); Jin *et al.* (2000 ▶, 2003 ▶, 2006 ▶); Juan *et al.* (1998 ▶); Kansikas *et al.* (1994 ▶); Li & Liu (2003 ▶); Rafizadeh *et al.* (2006 ▶); Terzis & Mentzafos (1983 ▶); Yousefi, Amani & Khavasi (2007 ▶); Yousefi, Ahmadi *et al.* (2007 ▶); Yousefi *et al.* (2007*a* ▶,*b* ▶); Zordan & Brammer (2004 ▶); Zordan *et al.* (2005 ▶).
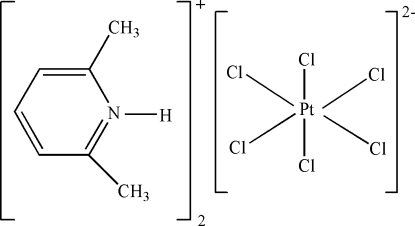


## Experimental

### 

#### Crystal data

(C_7_H_10_N)_2_[PtCl_6_]
*M* *_r_* = 624.10
Monoclinic, 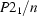

*a* = 9.9142 (12) Å
*b* = 9.6031 (10) Å
*c* = 11.3305 (14) Å
β = 107.117 (10)°
*V* = 1031.0 (2) Å^3^
*Z* = 2
Mo *K*α radiation
μ = 7.58 mm^−1^
*T* = 298 (2) K
0.48 × 0.45 × 0.38 mm

#### Data collection

Bruker SMART CCD area-detector diffractometer
Absorption correction: numerical (*X-SHAPE* and *X-RED*; Stoe & Cie, 2005 ▶)*T*_min_ = 0.41, *T*_max_ = 0.60
2756 measured reflections
2756 independent reflections
2387 reflections with *I* > 2σ(*I*)

#### Refinement

*R*[*F* ^2^ > 2σ(*F* ^2^)] = 0.069
*wR*(*F* ^2^) = 0.189
*S* = 1.10
2756 reflections
111 parameters
H atoms treated by a mixture of independent and constrained refinement
Δρ_max_ = 1.82 e Å^−3^
Δρ_min_ = −1.09 e Å^−3^

### 

Data collection: *SMART* (Bruker, 1998 ▶); cell refinement: *SAINT* (Bruker, 1998 ▶); data reduction: *SAINT*; program(s) used to solve structure: *SHELXTL* (Sheldrick, 2008 ▶); program(s) used to refine structure: *SHELXTL*; molecular graphics: *ORTEP-3 for Windows* (Farrugia, 1997 ▶); software used to prepare material for publication: *WinGX* (Farrugia, 1999 ▶).

## Supplementary Material

Crystal structure: contains datablocks I, global. DOI: 10.1107/S1600536808025257/hk2508sup1.cif

Structure factors: contains datablocks I. DOI: 10.1107/S1600536808025257/hk2508Isup2.hkl

Additional supplementary materials:  crystallographic information; 3D view; checkCIF report

## Figures and Tables

**Table:** 

Pt1—Cl2	2.3161 (16)
Pt1—Cl3	2.3239 (16)
Pt1—Cl1	2.3298 (14)

**Table:** 

Cl2—Pt1—Cl1	90.25 (6)
Cl2^i^—Pt1—Cl1	89.75 (6)
Cl2—Pt1—Cl3^i^	90.20 (8)
Cl2—Pt1—Cl3	89.80 (8)
Cl3—Pt1—Cl1^i^	89.37 (6)
Cl3—Pt1—Cl1	90.63 (6)

Symmetry code: (i) 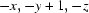.

**Table 2:** Hydrogen-bond geometry (Å, °)

*D*—H⋯*A*	*D*—H	H⋯*A*	*D*⋯*A*	*D*—H⋯*A*
N1—H1*D*⋯Cl3^ii^	0.85 (8)	2.45 (8)	3.279 (6)	168 (7)
C1—H1*B*⋯Cl1^ii^	0.96	2.83	3.654 (11)	145
C4—H4⋯Cl2^iii^	0.93	2.71	3.616 (11)	165

Symmetry codes: (ii) 
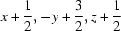
; (iii) 
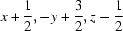
.

**Fig. 1.**
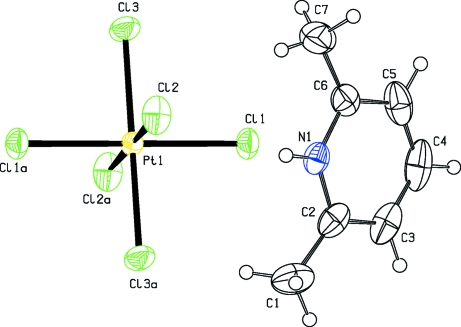
The molecular structure of the title molecule, with the atom-numbering scheme. Displacement ellipsoids are drawn at the 40% probability level [symmetry code: (a) -x, 1 - y, -z].

**Fig. 2.**
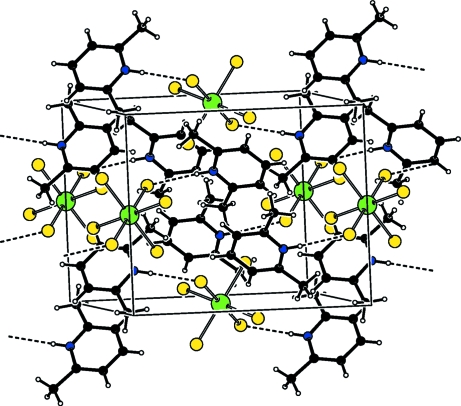
A packing diagram of (I). Hydrogen bonds are shown as dashed lines.

## supplementary crystallographic information

### Comment

In recent years, there has been considerable interest in proton transfer systems and their structures (Rafizadeh *et al.*,  2006; Yousefi, Amani & Khavasi, 2007; Abedi *et al.*,  2008; Hojjat Kashani *et al.*, 2008). Several proton transfer systems using 2,6-dimethylpyridine, with proton donor molecules, such as [2,6-dmpy.H](NO_3_), (II), (Jin *et al.*,  2003), [2,6-dmpy.H]_2_[CoCl_4_], (III), (Kansikas *et al.*,  1994), [2,6-dmpy.H]Cl, (IV), (Effendy *et al.*,  2006), [2,6-dmpy.H]_3_[BiBr_6_], (V), (Bowmaker *et al.*,  1998), [2,6-dmpy.H]_2_- [O_3_CrOCrO_3_], (VI), (Jin *et al.*,  2006) and [2,6-dmpy.H][Ph(COOH)(COO)], (VII), (Jin *et al.*,  2000) [2,6-dmpy.H is 2,6-dimethylpyridinium] have been synthesized and characterized by single-crystal X-ray diffraction methods.

There are also several proton transfer systems using H_2_[PtCl_6_] with proton acceptor molecules, such as [HpyBr-3]_2_[PtCl_6_].2H_2_O, (XIII), and [HpyI-3]_2_[PtCl_6_].2H_2_O, (IX),(Zordan & Brammer, 2004), [BMIM]_2_[PtCl_6_], (X), and [EMIM]_2_[PtCl_6_], (XI), (Hasan *et al.*,  2001), {(DABCO)H_2_[PtCl_6_]}, (XII), (Juan *et al.*,  1998), {*p*-C_6_H_4_(CH_2_ImMe)_2_[PtCl_6_]}, (XIII), (Li & Liu, 2003), [het][PtCl_6_].2H_2_O, (XIV), (Hu *et al.*, 2003), [9-MeGuaH]_2_[PtCl_6_].2H_2_O, (XV), (Terzis & Mentzafos, 1983), [H_10_[30]aneN_10_][PtCl_6_]_2_Cl_6_.2H_2_O, (XVI), (Bencini *et al.*, 1992), [H_2_Me_2_ppz][PtCl_6_], (XVII), (Ciccarese *et al.*,  1998), [PA]_2_[PtCl_6_]Cl, (XVIII), (Delafontaine *et al.*,  1987), [DEA]_2_[PtCl_6_], (XIX), (Bokach *et al.*,  2003), [HpyCl-3]_3_[PtCl_6_]Cl, (XX), (Zordan *et al.*,  2005), [2,9-dmphen.H]_2_- [PtCl_6_], (XXI), (Yousefi, Ahmadi *et al.*, 2007), [H_2_DA18C6][PtCl_6_].2H_2_O, (XXII), (Yousefi *et al.*,  2007*a*) and [TBA]_3_[PtCl_6_]Cl, (XXIII), (Yousefi *et al.*,  2007*b*) [where hpy is halo- pyridinium, BMI*M*^+^ is 1-*n*-butyl-3-methylimidazolium, EMI*M*^+^ is 1-ethyl-3-methylimidazolium, DABCO is 1,4-diazabicyclooctane, Im is imidazolium, het is 2-(α-hydroxyethyl) thiamine, 9-MeGuaH is 9-methylguaninium, [H_10_[30]aneN_10_] is [C_20_H_60_N_10_]^10+^ cation, H_2_Me_2_ppz is *N*,*N*'-dimethylpiperazinium, PA is pentane-1,5- diammonium, DEA is diethyl-ammonium, 2,9-dmphen.H is 2,9-dimethyl-1,10 -phenanthrolinium, H_2_DA18C6 is 1,10-Diazonia-18-crown-6 and TBA is tribenzylammonium] have been synthesized and characterized by single-crystal X-ray diffraction methods. We report herein the synthesis and crystal structure of the title compound, (I).

The asymmetric unit of (I), (Fig. 1) contains one independent protonated 2,6-di- methylpyridinium cation and half of a centrosymmetric [PtCl_6_]^2-^ anion. The Pt ion has an octahedral coordination. In cation, the bond lengths and angles are in good agreement with the corresponding values in (II) and (IV). In [PtCl_6_]^2-^ anion, the Pt-Cl bond lengths and Cl-Pt-Cl bond angles (Table 1) are also within normal ranges, as in (XXI), (XXII) and (XXIII).

In the crystal structure (Fig. 2), intermolecular N-H···Cl and C-H···Cl hydrogen bonds (Table 2) result in the formation of a supramolecular structure, in which they may be effective in the stabilization of the structure. A π—π contact between A (N1/C2-C6) rings Cg1···Cg1^i^ [symmetry code: (i) -x, 1 - y, 1 - z, where Cg1 is centroid of the ring A (N1/C2-C6)] further stabilize the structure, with centroid-centroid distance of 4.235 (1) Å.

### Experimental

For the preparation of the title compound, a solution of 2,6-dimethylpyridine (0.16 g, 1.48 mmol, 0.17 ml) in methanol (15 ml) was added to a solution of H_2_PtCl_6_.6H_2_O, (0.38 g, 0.74 mmol) in acetonitrile (15 ml) and the resulting yellow solution was stirred for 10 min at 313 K. Then, it was left to evaporate slowly at room temperature. After one week, orange prismatic crystals of were isolated (yield; 0.34 g; 73.6%).

### Refinement

H1D atom (for NH) was located in difference syntheses and refined isotropically [N-H = 0.85 (7) Å and U_iso_(H) = 0.029 (17) Å^2^]. The remaining H atoms were positioned geometrically, with C-H = 0.93 and 0.96 Å for aromatic and methyl H, respectively, and constrained to ride on their parent atoms with U_iso_(H) = 1.2U_eq_(C).

### Crystal data

**Table:**

(C_7_H_10_N)_2_[PtCl_6_]	*F*_000_ = 596
*M**_r_* = 624.10	*D*_x_ = 2.010 Mg m^−^^3^
Monoclinic, *P*2_1_/*n*	Mo *K*α radiation λ = 0.71073 Å
Hall symbol: -P 2yn	Cell parameters from 1071 reflections
*a* = 9.9142 (12) Å	θ = 2.4–29.1º
*b* = 9.6031 (10) Å	µ = 7.58 mm^−^^1^
*c* = 11.3305 (14) Å	*T* = 298 (2) K
β = 107.117 (10)º	Prism, orange
*V* = 1031.0 (2) Å^3^	0.48 × 0.45 × 0.38 mm
*Z* = 2	

### Data collection

**Table:**

Bruker SMART CCD area-detector diffractometer	2756 independent reflections
Radiation source: fine-focus sealed tube	2387 reflections with *I* > 2σ(*I*)
Monochromator: graphite	*R*_int_ = 0.094
*T* = 298(2) K	θ_max_ = 29.1º
φ and ω scans	θ_min_ = 2.4º
Absorption correction: numerical(X-SHAPE and X-RED; Stoe & Cie, 2005)	*h* = −13→13
*T*_min_ = 0.41, *T*_max_ = 0.60	*k* = −12→13
2756 measured reflections	*l* = −15→15

### Refinement

**Table:**

Refinement on *F*^2^	Hydrogen site location: inferred from neighbouring sites
Least-squares matrix: full	H atoms treated by a mixture of independent and constrained refinement
*R*[*F*^2^ > 2σ(*F*^2^)] = 0.069	*w* = 1/[σ^2^(*F*_o_^2^) + (0.1499*P*)^2^ + 0.5352*P*] where *P* = (*F*_o_^2^ + 2*F*_c_^2^)/3
*wR*(*F*^2^) = 0.189	(Δ/σ)_max_ = 0.019
*S* = 1.10	Δρ_max_ = 1.82 e Å^−^^3^
2756 reflections	Δρ_min_ = −1.09 e Å^−^^3^
111 parameters	Extinction correction: SHELXTL (Sheldrick, 2008), Fc^*^=kFc[1+0.001xFc^2^λ^3^/sin(2θ)]^-1/4^
Primary atom site location: structure-invariant direct methods	Extinction coefficient: 0.029 (3)
Secondary atom site location: difference Fourier map	

### Special details

**Table:**

Experimental. shape of crystal determined optically (X-SHAPE and X-RED; Stoe & Cie, 2005)
Geometry. All e.s.d.'s (except the e.s.d. in the dihedral angle between two l.s. planes) are estimated using the full covariance matrix. The cell e.s.d.'s are taken into account individually in the estimation of e.s.d.'s in distances, angles and torsion angles; correlations between e.s.d.'s in cell parameters are only used when they are defined by crystal symmetry. An approximate (isotropic) treatment of cell e.s.d.'s is used for estimating e.s.d.'s involving l.s. planes.
Refinement. Refinement of F^2^ against ALL reflections. The weighted R-factor wR and goodness of fit S are based on F^2^, conventional R-factors R are based on F, with F set to zero for negative F^2^. The threshold expression of F^2^ > 2sigma(F^2^) is used only for calculating R-factors(gt) etc. and is not relevant to the choice of reflections for refinement. R-factors based on F^2^ are statistically about twice as large as those based on F, and R- factors based on ALL data will be even larger.

### Fractional atomic coordinates and isotropic or equivalent isotropic displacement parameters (Å^2^)

**Table:**

	*x*	*y*	*z*	*U*_iso_*/*U*_eq_	
Pt1	0.0000	0.5000	0.0000	0.0265 (2)	
Cl1	0.14887 (17)	0.56949 (18)	−0.11435 (15)	0.0411 (4)	
Cl2	0.0667 (2)	0.6927 (2)	0.12679 (17)	0.0504 (5)	
Cl3	−0.18455 (17)	0.6275 (2)	−0.12863 (15)	0.0486 (5)	
N1	0.3768 (6)	0.8089 (7)	0.1075 (5)	0.0409 (12)	
H1D	0.348 (8)	0.822 (8)	0.170 (7)	0.029 (17)*	
C1	0.5069 (11)	0.6118 (11)	0.2190 (9)	0.069 (2)	
H1A	0.4229	0.5663	0.2249	0.082*	
H1B	0.5475	0.6653	0.2924	0.082*	
H1C	0.5735	0.5431	0.2100	0.082*	
C2	0.4710 (8)	0.7054 (9)	0.1103 (8)	0.0503 (18)	
C3	0.5217 (11)	0.6928 (17)	0.0112 (9)	0.062 (3)	
H3	0.5875	0.6241	0.0104	0.074*	
C4	0.4756 (12)	0.7819 (14)	−0.0875 (10)	0.076 (3)	
H4	0.5090	0.7727	−0.1557	0.091*	
C5	0.3790 (11)	0.8854 (11)	−0.0849 (7)	0.064 (3)	
H5	0.3482	0.9462	−0.1513	0.077*	
C6	0.3278 (8)	0.8987 (8)	0.0163 (7)	0.0472 (16)	
C7	0.224 (2)	1.0036 (8)	0.033 (2)	0.071 (5)	
H7A	0.1375	0.9938	−0.0325	0.085*	
H7B	0.2617	1.0955	0.0302	0.085*	
H7C	0.2072	0.9892	0.1109	0.085*	

### Atomic displacement parameters (Å^2^)

**Table:**

	*U*^11^	*U*^22^	*U*^33^	*U*^12^	*U*^13^	*U*^23^
C1	0.057 (5)	0.061 (5)	0.073 (6)	0.009 (4)	−0.003 (4)	−0.008 (4)
C2	0.034 (3)	0.053 (4)	0.060 (4)	−0.009 (3)	0.009 (3)	−0.022 (3)
C3	0.046 (4)	0.071 (7)	0.076 (7)	−0.015 (5)	0.028 (4)	−0.031 (5)
C4	0.069 (6)	0.111 (9)	0.061 (5)	−0.038 (6)	0.041 (5)	−0.029 (6)
C5	0.067 (5)	0.086 (6)	0.036 (3)	−0.035 (5)	0.011 (4)	0.001 (4)
C6	0.040 (3)	0.051 (4)	0.044 (3)	−0.013 (3)	0.003 (3)	0.001 (3)
C7	0.060 (10)	0.054 (8)	0.092 (15)	−0.002 (3)	0.011 (10)	0.016 (4)
N1	0.039 (3)	0.053 (3)	0.034 (2)	−0.005 (2)	0.016 (2)	−0.007 (2)
Pt1	0.0244 (3)	0.0322 (3)	0.0223 (3)	−0.00137 (8)	0.00597 (18)	0.00008 (7)
Cl1	0.0382 (8)	0.0514 (9)	0.0389 (8)	−0.0048 (6)	0.0196 (6)	0.0033 (6)
Cl2	0.0563 (10)	0.0491 (9)	0.0502 (9)	−0.0189 (7)	0.0224 (8)	−0.0207 (7)
Cl3	0.0365 (8)	0.0688 (11)	0.0390 (8)	0.0165 (7)	0.0087 (6)	0.0169 (7)

### Geometric parameters (Å, °)

**Table:**

Pt1—Cl2	2.3161 (16)	C2—C3	1.365 (11)
Pt1—Cl2^i^	2.3161 (16)	C3—C4	1.374 (19)
Pt1—Cl3^i^	2.3239 (16)	C3—H3	0.9300
Pt1—Cl3	2.3239 (16)	C4—C5	1.387 (18)
Pt1—Cl1^i^	2.3298 (14)	C4—H4	0.9300
Pt1—Cl1	2.3298 (14)	C5—C6	1.390 (12)
N1—H1D	0.85 (7)	C5—H5	0.9300
C1—C2	1.480 (14)	C6—N1	1.323 (10)
C1—H1A	0.9600	C6—C7	1.49 (2)
C1—H1B	0.9600	C7—H7A	0.9600
C1—H1C	0.9600	C7—H7B	0.9600
C2—N1	1.357 (10)	C7—H7C	0.9600
			
Cl1^i^—Pt1—Cl1	180.00 (8)	H1B—C1—H1C	109.5
Cl2—Pt1—Cl1^i^	89.75 (6)	N1—C2—C3	117.6 (10)
Cl2^i^—Pt1—Cl1^i^	90.25 (6)	N1—C2—C1	117.4 (8)
Cl2—Pt1—Cl1	90.25 (6)	C3—C2—C1	125.0 (10)
Cl2^i^—Pt1—Cl1	89.75 (6)	C2—C3—C4	120.0 (12)
Cl2—Pt1—Cl2^i^	180.00 (6)	C2—C3—H3	120.0
Cl2—Pt1—Cl3^i^	90.20 (8)	C4—C3—H3	120.0
Cl2^i^—Pt1—Cl3^i^	89.80 (8)	C3—C4—C5	119.7 (9)
Cl2—Pt1—Cl3	89.80 (8)	C3—C4—H4	120.2
Cl2^i^—Pt1—Cl3	90.20 (8)	C5—C4—H4	120.2
Cl3^i^—Pt1—Cl1^i^	90.63 (6)	C4—C5—C6	120.4 (9)
Cl3—Pt1—Cl1^i^	89.37 (6)	C4—C5—H5	119.8
Cl3^i^—Pt1—Cl3	180.0	C6—C5—H5	119.8
Cl3^i^—Pt1—Cl1	89.37 (6)	N1—C6—C5	116.4 (8)
Cl3—Pt1—Cl1	90.63 (6)	N1—C6—C7	116.9 (11)
C6—N1—C2	126.0 (7)	C5—C6—C7	126.7 (12)
C6—N1—H1D	115 (5)	C6—C7—H7A	109.5
C2—N1—H1D	119 (5)	C6—C7—H7B	109.5
C2—C1—H1A	109.5	H7A—C7—H7B	109.5
C2—C1—H1B	109.5	C6—C7—H7C	109.5
H1A—C1—H1B	109.5	H7A—C7—H7C	109.5
C2—C1—H1C	109.5	H7B—C7—H7C	109.5
H1A—C1—H1C	109.5		
			
N1—C2—C3—C4	−1.0 (14)	C4—C5—C6—C7	−179.9 (12)
C1—C2—C3—C4	176.8 (10)	C5—C6—N1—C2	−0.5 (11)
C2—C3—C4—C5	0.9 (15)	C7—C6—N1—C2	179.7 (10)
C3—C4—C5—C6	−0.5 (14)	C3—C2—N1—C6	0.8 (11)
C4—C5—C6—N1	0.3 (11)	C1—C2—N1—C6	−177.1 (7)

Symmetry codes: (i) −*x*, −*y*+1, −*z*.

### Hydrogen-bond geometry (Å, °)

**Table:**

*D*—H···*A*	*D*—H	H···*A*	*D*···*A*	*D*—H···*A*
N1—H1D···Cl3^ii^	0.85 (8)	2.45 (8)	3.279 (6)	168 (7)
C1—H1B···Cl1^ii^	0.96	2.83	3.654 (11)	145
C4—H4···Cl2^iii^	0.93	2.71	3.616 (11)	165

Symmetry codes: (ii) *x*+1/2, −*y*+3/2, *z*+1/2; (iii) *x*+1/2, −*y*+3/2, *z*−1/2.
